# A case of bilateral pleural effusion as the first sign of multiple myeloma

**DOI:** 10.1186/2047-783X-18-7

**Published:** 2013-03-20

**Authors:** Xuan-li Xu, Yi-hong Shen, Qian Shen, Jian-ying Zhou

**Affiliations:** 1 Department of Respiratory Medicine, the First Affiliated Hospital, School of Medicine, Zhejiang University, 79 Qingchun Road, Hangzhou, 310003, China

**Keywords:** Multiple myeloma, Pleural effusion

## Abstract

Multiple myeloma (MM) is a type of hematological malignancy that can affect all types of tissues in human. However, it is extremely rare that pleural effusion presents as the first sign in MM patients. A 54-year-old male patient attended our department of respiratory medicine complaining of shortness of breath for the past 3 months. A chest computer tomography (CT) radiograph revealed a bilateral pleural effusion, which was further assessed as exudative type. Sinus spiral CT scan demonstrated diffuse bone destruction of craniofacial bone. A broad reduction of the lumbar bone signal was confirmed by MRI. Furthermore, pleural biopsy showed abnormal proliferation of plasmocytes whereas bone marrow biopsy showed active hyperplasia of plasmacytoid cells. Interestingly, Bence-Jones protein in urine and serum protein electrophoresis was negative. The patient was diagnosed as non-secretory MM. He then underwent chemotherapy with vincristine, adriamycin and dexamethasone. Partial regression of the pleural effusion was achieved after two rounds of chemotherapy, and the patient has been followed for more than one year.

## Background

Multiple myeloma (MM) is one of the most common hematological malignancies; it is a malignant plasma cell neoplasm that affects over 20,000 people yearly in the United States [1]. MM affects a broad range of tissues, while mainly in the bone marrow. It is particularly rare (less than 1%) for MM patients to present with pleural effusion caused by abnormal proliferation of plasma cells [2]. Pleural effusion in MM normally indicates a poor prognosis for myeloma and a low possibility of survival exceeding 4 months. Here, we report a case of MM with pleural effusion as the first presenting sign. The pleural nodular-like thickening of the thorax was very distinctive with CT imaging. Furthermore, a dramatically elevated adenosine deaminase (ADA) activity was also observed in the pleural fluid.

## Case presentation

A 54-year-old man was admitted to our department due to the dyspnea on exertion for more than 3 months. The illness began in March 2011, presenting as dyspnea on exertion and relieved by rest accompanied by mild reduced weight. The patient had no fever, no cough, no chest pain or palpitations. His previous history revealed cervical spondylosis, left side waist and leg pain for 20 years, and recurrent sinusitis and otitis media for 6 months. No special consideration was found in his personal or family history, except for a 90 packs-a-year smoking history. On physical examination, his vital signs were as follows: blood pressure: 119/65 mmHg; heart rate: 68 beats/minute; respiratory rate: 18 breaths/minute; temperature: 36.8°C. The respiratory examination indicated decreased breath sounds in the bilateral lower hemithorax, with dullness on percussion.

Laboratory results revealed the following values: white blood cell (WBC) count: 3.4 × 10^3^/μL (59% neutrophils, 35% lymphocytes, 3.9% monocytes, 1.7% eosinophils and 0.4% basophils); hemoglobin: 6.8 g/dL; platelet count: 156 × 10^3^/μL; total protein: 6.1 g/dL (normal range: 6.0 to 8.3 g/dL); albumin: 4.1 g/dL (normal range: 3.5 to 5.5 g/dL); globulin: 2.0 g/dL(normal range: 2.0-3.5 g/dL); ADA 175U/L(normal range: 0 to 18U/L); uric acid 608 μmol/L(normal range: 90 to 420 μmol/L); erythrocyte sedimentation rate (ESR): 102 mm/h; CA-125: 225.4U/mL (normal range: 0 to 35 U/mL); ferritin: 375 ng/mL (normal range: 7 to 323 ng/mL); purified protein derivative (PPD): negative. Bence-Jones proteins in urine and serum protein electrophoresis were negative. The serum immunoglobulin A (22 mg/dL), -M (18 mg/dL), -G (535 mg/dL) levels were all not noteworthy, also were the serum Kappa (130 mg/dL) and lambda light chain levels (126 mg/dL).

Analysis of the right side pleural fluid obtained by thoracentesis indicated an exudative type. The pleural fluid contained WBC 2250/μL (47% neutrophils, 23% lymphocytes, 30% mesothelial cells), total protein 3.3 g/dL, Rivalta test ++, LDH 138 IU/L, and a drastically elevated total ADA 419.0U/L (normal range: <45 IU/L). No acid-resistant bacilli were detected in the pleural fluid. The left side pleural fluid showed the similar exudative type, but almost no mesothelial cells.

**Figure 1:**
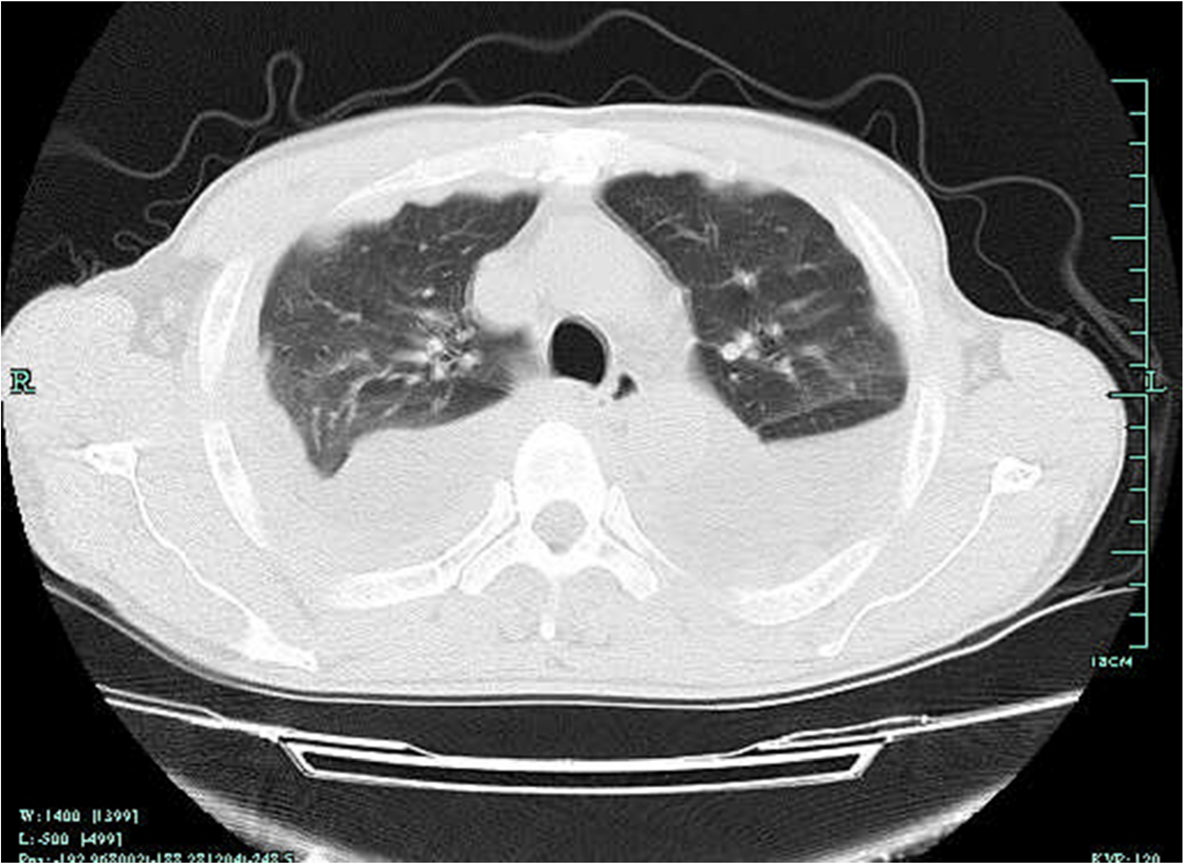
**CT scan of the thorax.** CT scan of the thorax showing a bilateral pleural effusion, diffused pleural thickening and multiple nodular lesions in the parietal pleura.

**Figure 2:**
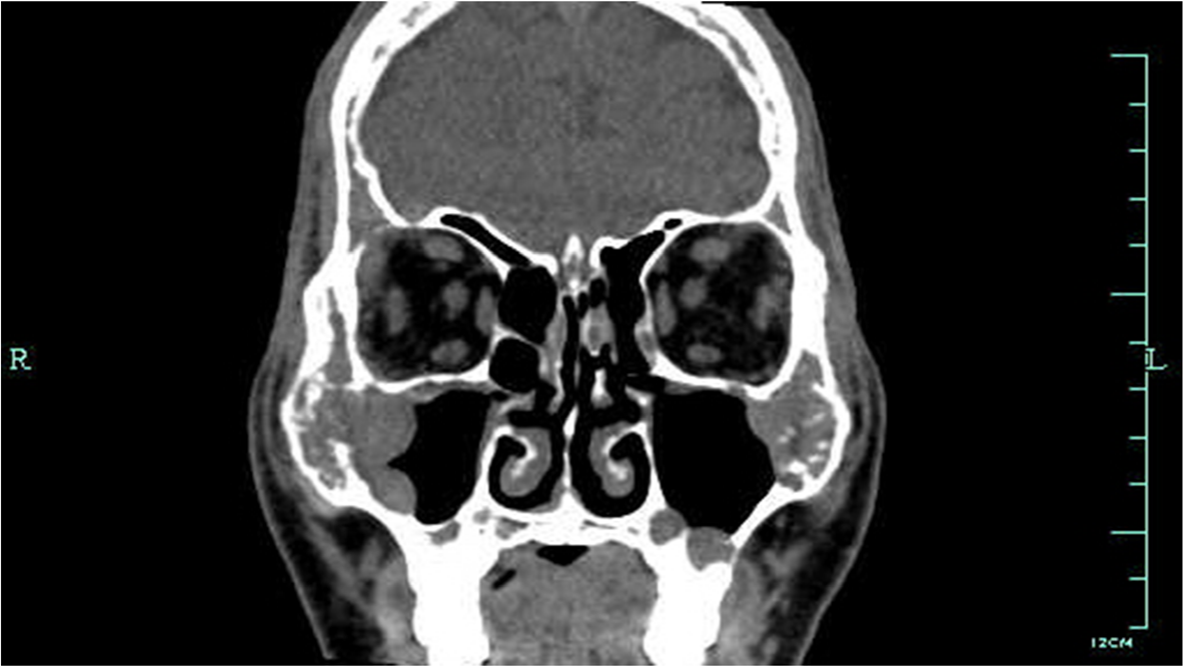
**Computed tomography of the sinus spiral.** Computed tomography of the sinus spiral showing a pansinusitis and diffuse bone destruction of craniofacial bone.

**Figure 3:**
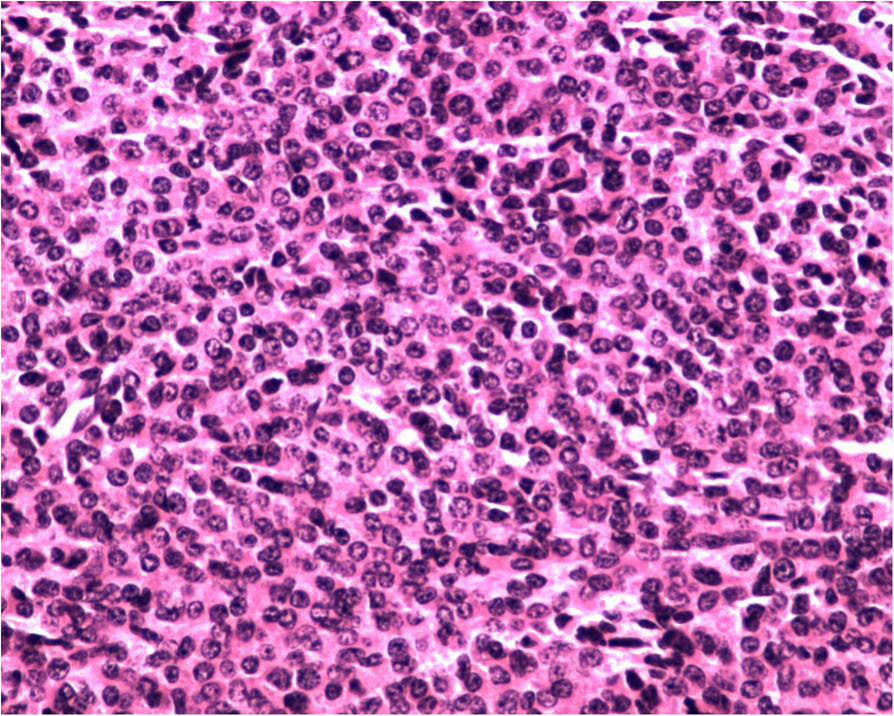
**Bone marrow biopsy.** Abundant atypical plasmacytoid cells were detected in the bone marrow biopsy (magnification 400×).

**Figure 4:**
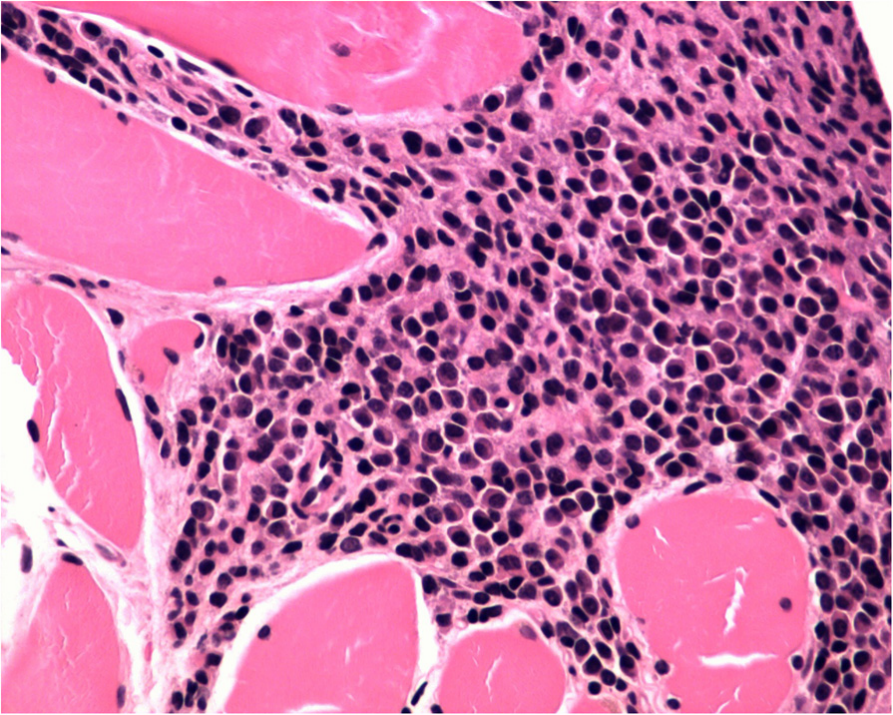
**Pleural biopsy.** Histological findings of the pleural biopsy showing abnormal proliferation of plasmocytes (magnification 400×).

A CT imaging of the chest showed bilateral pleural effusion, with more pleural fluid in the right side. Bilateral diffused pleural thickening, and strikingly, multiple nodular lesions in the parietal pleura, bilateral lower lung segmental atelectasis, but no mediastinal or hilaradenopathy, was observed in the CT images (Figure 1). The sinus spiral CT demonstrated pansinusitis and diffuse bone destruction of craniofacial bone (Figure 2). Then, MRI was further performed because of his previous history of left lumbar and leg pain. Consistently, the lumbar MRI showed a broad reduced signal of the lumbar bone indicating a hematopoietic disease. The lytic lesions were further demonstrated by emission computed tomography (ECT) of the total skeletal system. The bone marrow biopsy revealed active hyperplasia of plasma cell (76%) and lymphoid plasma cells (Figure 3). A biopsy of the right side pleura guided by CT further confirmed abnormal proliferation of plasmocytes (Figure 4). The biopsy of the left was not performed because of the unobvious pleural nodules and less effusion.

The patient was diagnosed with non-secretory MM. He was transferred to Department of Hematology, and underwent chemotherapy with vincristine, adriamycin and dexamethasone. After two rounds of chemotherapy, partial regression of pleural effusion, and pain relief was achieved. Currently, the patient is still alive and has been followed for more than one year since then.

## Conclusions

Myeloma is a very rare cause of malignant pleural effusion. The presentation in this case was unique in regards to the negative serum and urine protein electrophoresis when the patient initially presented with a bilateral plasmacytic pleural effusion in the respiratory clinical. In addition, it is also unusual that dramatically elevated ADA activity presented in his pleural effusion fluid.

Pleural effusion in MM may be due to a variety of malignant and non-malignant reasons, such as congestive heart failure, pneumonia, tuberculosis, carcinomatosis, hypoproteinemia and other viral illness. For this case, tuberculosis and pleural mesothelioma should be differentially diagnosed because of his highly elevated ADA activity in effusion fluid and multiple nodular lesions in the parietal pleura.

Sasser *et al*. showed 56 cases of MM with the involvement of the serous cavities. The sites of involvement included the pleural cavity in 30 cases, among which, over 50% of the cases with cavitary involvement were of the IgA type [3]. Involvement of the bilateral pleural effusion by multiple myeloma is extremely rare. So far, only two similar cases have been reported with bilateral immature plasma cells pleural involvement [4,5].

Several possible mechanisms are postulated for myelomatous pleural effusion. The first possibility is heart failure but not the tumorous violation. Kintzer *et al*. reported that about half of MM patients accompanied with pleural effusion were those with congestive heart failure [6]. It is possible that heart failure maybe caused by cardiac amyloidosis derived from MM. The other possible mechanisms are the pulmonary embolism, chronic renal failure accompanied with MM and second tumor. Thus, our case is particularly distinctive in regards to the direct violation of the malignant plasma cells to the pleura, as confirmed with the biopsy.

We also observed a drastically elevated ADA activity (419.0U/L) in the pleural fluid in this case. Our initial tentative diagnosis was tuberculosis, according to the high level of ADA and the negative Bence-Jones protein in urine and serum protein electrophoresis. The increased ADA was commonly found in the tuberculous pleural fluid. Nevertheless, an elevated ADA activity in pleural fluid has also been reported in some cases of benign or malignant diseases, such non-Hodgkin’s lymphoma, breast cancer [7,8]. Yokoyama *et al.* also reported a myelomatous pleural effusion case with high ADA activity in pleural effusion [9], but the ADA activity in our patient was much higher.

Although it is atypical that this MM case initially presented with bilateral pleural effusion, clues can still be identified from detailed previous history and laboratory tests: i) Infections: acute bacterial infection caused by MM could be the first manifestation, the main complication, and the main cause of death. A great amount of M protein produced in MM may inhibit the normal immunoglobulin synthesis, thus leading to immune deficiency. Consistently, this patient had recurrent sinusitis and otitis media; ii) Anemia: anemia is a common clinical manifestation of MM, mainly due to malignant tumor cell proliferation in the bone marrow. For this case, the patient had moderate normocytic normochromic anemia;iii) Bone destruction: the main X-ray presentations of MM are osteolytic bone destruction, extensive osteoporosis and pathological fractures. CT and MRI scan of this patient showed extensive destruction of the skull and spine.

In summary, this case report suggests that bilateral pleural effusion accompanied with multiple nodular lesions in pleura and high ADA activity in pleural effusion fluid may be caused by MM. The presence of atypical plasma cells in the pleura may provide useful evidence for the diagnosis of MM. Myelomatous pleural effusion as an initial presentation, although extremely rare, should always be considered in presence of atypical plasma cells and multiple nodular lesions in pleura.

## Consent

Written informed consent was obtained from the patient for publication of this case report and any accompanying images. A copy of the written consent is available for review by the Editor-in-Chief of this journal.

## Abbreviations

ADA: Adenosine deaminase; ECT: Emission computed tomography; MM: Multiple myeloma; PPD: Purified protein derivative; WBC: White blood cell.

## Competing interests

The authors declare that they have no competing interests.

## Authors’ contributions

XX, study concept and design, acquisition of data, analysis and interpretation of data, drafting of the manuscript; YS, technical or material support; QS, technical or material support; JZ, critical revision of the manuscript for important intellectual content. All authors read and approved the final manuscript.

## Authors’ information

Xuan-li Xu, doctor’s degree, physician; Yi-hong Shen, doctor’s degree, attending physician; Qian Shen, master’s degree, physician; Jian-ying Zhou, master’s degree, professor, attending physician, director of respiratory medicine.
